# Exocytosed ATP as a therapeutic target for inflammatory and metabolic diseases

**DOI:** 10.3389/fphar.2026.1856512

**Published:** 2026-07-01

**Authors:** Nao Hasuzawa, Seiji Nomura, Sawako Moriyama, Yoshinori Moriyama, Masatoshi Nomura

**Affiliations:** 1 Division of Endocrinology and Metabolism, Department of Internal Medicine, Kurume University School of Medicine, Kurume, Japan; 2 Department of Medical Biochemistry, Kurume University School of Medicine, Kurume, Japan

**Keywords:** ATP exocytosis, chronic inflammation, exocytosed ATP, metabolic disorders, pain, purinergic signaling, SLC17A9, vesicular nucleotide transporter (VNUT)

## Abstract

Extracellular adenosine triphosphate (eATP) is a key intercellular signaling molecule in purinergic transmission and contributes to pain, inflammation, and tissue dysfunction in a wide range of diseases. Although eATP is released through membrane damage, conductive pathways, and vesicular exocytosis, the biological and pharmacological significance of ATP released by exocytosis has remained insufficiently defined. Vesicular nucleotide transporter (VNUT), encoded by SLC17A9, which loads ATP into secretory vesicles, is essential for ATP exocytosis and has enabled direct investigation of the roles of exocytosed ATP in physiology and disease. Accumulating evidence indicates that VNUT-dependent ATP exocytosis contributes to inflammatory signaling, sensory transmission, metabolic dysregulation, and cancer-related processes, while its inhibition shows therapeutic potential in experimental models of pathological states. This review summarizes recent advances in the molecular basis, regulation, pharmacological manipulation, and disease relevance of VNUT-mediated ATP exocytosis. We also discuss the therapeutic potential of targeting exocytosed ATP for pain, chronic inflammation, and metabolic disorders.

## 1 Introduction

In 1941, Fritz Lipmann recognized the crucial role of the phosphate bond energy at the γ position of ATP in cellular metabolism. He discovered that enzymatic reactions often rely on the transfer of the γ-phosphate from ATP and established ATP’s significance as the universal energy currency (Lipmann et al., 1941). Since then, understanding the mechanism of oxidative phosphorylation—how cells generate ATP—has become a central focus of 20th-century research and has been largely clarified. Following Lipmann’s report, F.A. Holton and P. Holton at the University of Cambridge discovered that electrically stimulating rabbit auricular nerves produced a substance resembling ATP in the perfusion fluid. This finding suggested that extracellular ATP (eATP) may play a role in nerve-to-muscle cell transmission (Holton and Holton, 1954). Building on this, Geoffrey Burnstock demonstrated that the signaling molecules involved in this process are, in fact, ATP released from nerves and its metabolites, thereby confirming the existence of intercellular signaling through eATP, now known as purinergic chemical transmission (Burnstock, 1972). As research continues, the biological and medical significance of eATP is becoming increasingly evident, establishing the study of purinergic chemical transmission as a crucial scientific pursuit in the 21st century.

Purinergic chemical transmission, similar to classical neurotransmitter systems like acetylcholine and L-glutamate, involves the release of ATP into the extracellular space (signal output), subsequent signal reception and transduction via receptors (signal input), and signal termination. However, purinergic signaling exhibits distinct characteristics not typically observed in other extracellular signaling systems. First, eATP as an intercellular transmitter is notable for its rapid and sequential breakdown by extracellular ectonucleotidases into ADP, AMP, and finally adenosine, each step relaying information through specific purinoceptors. Ultimately, adenosine is either taken up by equilibrative nucleoside transporters 1 and 2 (ENT1/2) or metabolized into inosine and ammonia by adenosine deaminase, thereby reducing transmitter concentration near the receptor and terminating the signal. This continuous metabolic turnover of purines, both intracellularly and extracellularly, is a hallmark of purinergic chemical transmission (Burnstock, 2007; Abbracchio et al., 2009; Eltzschig, 2009).

Extracellular ATP and its metabolites primarily signal through P2 receptors, which are divided into ionotropic P2X receptors and metabotropic P2Y receptors, whereas its degradation product adenosine activates P1 receptors. P2X receptors are ATP-gated cation channels, while P2Y and P1 receptors are G protein-coupled receptors that regulate diverse intracellular signaling pathways. Through this receptor network, extracellular purines coordinate inflammation, neurotransmission, immunity, and tissue homeostasis (Huang et al., 2021).

**FIGURE 1:**
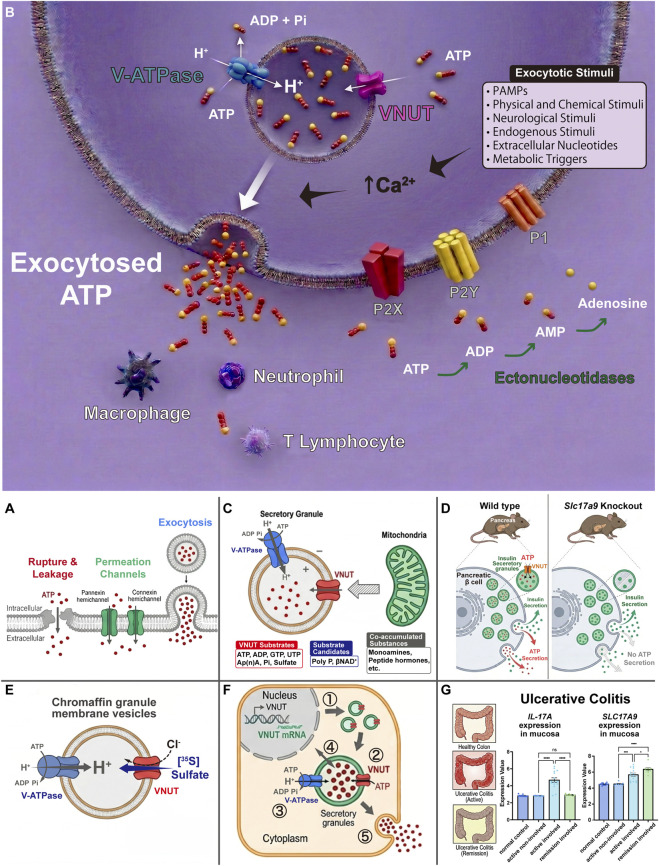
Mechanisms and clinical relevance of VNUT-mediated ATP exocytosis. **(A)** Three major pathways of ATP release into the extracellular space (Lipmann et al., 1941): rupture/leakage (Holton and Holton, 1954), channel-mediated permeation (e.g., connexin and pannexin hemichannels), and (Burnstock, 1972) vesicular exocytosis. **(B)** Schematic model of ATP exocytosis. VNUT actively transports ATP into secretory vesicles using the electrochemical potential difference generated by V-ATPase. In response to extracellular or intracellular stressors indicated as exocytotic stimuli in the diagram, elevated intracellular Ca^2+^ concentrations trigger the exocytosis of ATP-containing vesicles. The exocytosed ATP is metabolized to ADP, AMP, and adenosine. These purines transmit signals via purinoceptors. An autocrine mode of signaling is illustrated. **(C)** Mechanism of substrate accumulation into secretory vesicles. VNUT utilizes the membrane potential and proton gradient generated by V-ATPase to transport substrates. In addition to ATP, VNUT transports other nucleotides (ADP, GTP, UTP, and Ap(n)A), inorganic phosphate (Pi), and sulfate. Mitochondria provide ATP and other substrates, thereby influencing the contents of VNUT-containing vesicles. PolyP and βNAD^+^ are shown as candidate substrates, whereas monoamines and peptide hormones are shown as co-accumulated substances. **(D)** Essential role of VNUT in ATP exocytosis. Schematic model of the essential role of VNUT in ATP exocytosis. In wild-type mice, VNUT localizes to insulin secretory granules in pancreatic β-cells and is required for vesicular storage of ATP along with insulin. In *Slc17a9* knockout mice, ATP is not loaded into insulin granules, resulting in the loss of ATP exocytosis, whereas insulin secretion is preserved. Deletion or inhibition of VNUT abolishes or reduces exocytosed ATP, thereby providing a means to selectively suppress ATP exocytosis. Based on Sakamoto et al. (Sakamoto et al., 2014). **(E)** *In vitro* assay for quantifying VNUT activity. Isolated secretory granules (e.g., chromaffin granules) take up radiolabeled sulfate when ATP is supplied to generate a proton motive force via V-ATPase. Although Cl^−^ is not an essential component for the transport, extravesicular Cl^−^ acts as a regulatory factor that modulates the rate of VNUT-mediated sulfate uptake. **(F)** Potential molecular targets for regulating ATP exocytosis: (Lipmann et al., 1941) transcription, translation, and intracellular transport of VNUT; (Holton and Holton, 1954) VNUT transport activity; (Burnstock, 1972) V-ATPase-driven proton transport; (Burnstock, 2007) the ion and proton permeability of the vesicular membrane; and (Abbracchio et al., 2009) the machinery involved in exocytotic membrane fusion, such as SNAREs. **(G)** Mucosal *IL-17A* and *SLC17A9* expression in Ulcerative Colitis (UC). Gene expression values for *IL17A* and *SLC17A9* were extracted from the public microarray dataset GSE38713 and compared among healthy controls (normal control, n = 13), non-involved (n = 7) and involved (n = 15) mucosa from patients with active UC, and involved mucosa from patients with UC in remission (n = 8). Statistics: Mean ± SEM; one-way ANOVA followed by Sidak’s multiple comparisons test (*p < 0.0332, ***p < 0.0002, ****p < 0.0001). Data source: GSE38713 (Planell et al., 2013).

The extracellular supply of ATP possesses unique characteristics that distinguish it from most chemical signaling pathways. While many pathways primarily rely on exocytosis, ATP is released into the extracellular space through three distinct mechanisms (Figure 1A). The first mechanism, known as “leakage,” occurs when the cell membrane is compromised by physical, chemical, or biological damage. Because mammalian cytoplasm contains 2–4.4 mM ATP, membrane damage results in a large release of ATP, which serves as a “find me” signal to attract immune cells—such as neutrophils and macrophages—to the site of injury (Di Virgilio et al., 2020; Ferrari et al., 2021; Eltzschig et al., 2012). This process is the basis for the classification of eATP as a danger-associated molecular pattern (DAMP).

The second major pathway involves ATP permeation through various membrane channels (Di Virgilio et al., 2020; Ferrari et al., 2021; Eltzschig et al., 2012; Sabirov and Okada, 2005). Connexin hemichannels and pannexin channels play an important role in inflammatory responses (Adamson and Leitinger, 2014; Makarenkova and Shestopalov, 2014). These pore-forming channels mediate the release of small molecules in the extracellular space, including ATP, glutamate, and prostaglandins, as well as the influx of ions such as Na^+^ and Ca^2+^ (Lohman and Isakson, 2014). Their gating is regulated by transmembrane voltage, extracellular or intracellular calcium concentrations, mechanical stress or post-translational modifications (Dosch et al., 2018).

The third pathway is exocytosis. The hypothesis that exocytosis mediates eATP production was proposed early on, when eATP was first discovered. For ATP to be released via exocytosis, it must be packaged into secretory vesicles. Although several ATP-filled organelles have been identified (Zimmermann, 1994; Zimmermann, 2008), this pathway was far less characterized for many years because a transporter capable of supplying ATP to secretory vesicles had not been identified, and measuring such activity proved difficult. In 2008, the vesicular nucleotide transporter (VNUT) was finally identified as a key component of the vesicular ATP filling (Sawada et al., 2008; Moriyama et al., 2017). This discovery filled the final gap in our understanding of ATP exocytosis, enabling detailed analysis of the effects of exocytosed ATP, i.e., eATP produced via the exocytosis pathway. As research into VNUT function has advanced, it has become evident that exocytosed ATP is intimately involved in nearly all physiological processes regulated by purinergic chemical transmission, including infection, sensory transmission, and the progression of inflammation. Notably, targeting VNUT enables quantitative regulation of eATP levels. Due to its roles in cellular function, its associations with disease, and its potential as a therapeutic target, VNUT is now recognized as an influential player in a wide range of biomedical fields, including inflammation (Dosch et al., 2018; Hasuzawa et al., 2020).

Extracellular ATP signaling regulates a wide range of physiological and pathological processes through the coordinated interplay of ATP release, ectonucleotidase-mediated degradation, and purinergic receptor activation. In this review, we focus on eATP release as an upstream event in this cascade, with particular emphasis on ATP exocytosis, and discuss its physiological and pathological roles, particularly in light of recent VNUT-related research. We propose that VNUT-dependent ATP exocytosis represents a quantitatively controllable node in purinergic signaling, distinct from leakage- or channel-mediated ATP release. Among potential targets for modulating eATP signaling—ATP release, purinergic receptors, and ectonucleotidase activity—, targeting ATP exocytosis represents one possible strategy for upstream modulation of this complex network.

## 2 VNUT as a molecular basis for ATP exocytosis

### 2.1 VNUT is essential for ATP exocytosis

Filling secretory vesicles with ATP marks the first step in ATP exocytosis and an essential upstream step in purinergic chemical transmission. This process depends on the active transport of ATP, which is achieved through the functional coupling of VNUT and vacuolar H^+^-ATPase (V-ATPase) (Figures 1B,C). The vesicular nucleotide transporter (VNUT), encoded by *SLC17A9* in humans and *Slc17a9* in rodents, is the ninth member of the type I Na^+^-dependent phosphate transporter family (Moriyama et al., 2017). In this review, we use *SLC17A9*/*Slc17a9* when referring to genes and VNUT when referring to the protein/transporter. This human membrane protein consists of 430 amino acids and spans the membrane with 12 transmembrane domains. Unlike other SLC17 members, VNUT is highly hydrophobic, with minimal hydrophilic regions at both its amino and carboxyl termini. Two splicing variants, commonly referred to as VNUT1 and VNUT2, have been identified.

The biochemical properties of VNUT have been studied in humans, mice, cattle, and *Drosophila.* Within cells, VNUT actively transports MgATP as a divalent anion into vesicles using the Δψ (positive inside) generated by the V-ATPase as the driving force. The transport K_m_ for MgATP is approximately 0.8–2 mM. Under ideal conditions, such as Δψ = 80 mV, ATP can be concentrated to approximately 200 mM within the vesicle, according to the equation RT ln[ATPin]/[ATPout] = 2FΔψ (R = gas constant, T = absolute temperature, F = Faraday constant) (Omote et al., 2011). Under physiological conditions, ATP concentrations vary widely across organelles, ranging from several-fold to several hundred-fold higher than in the cytosol. VNUT transports ATP at a rate about 1/1,000 that of ion channels, but the high concentration of ATP within the vesicle enables rapid, directional release via exocytosis, leading to highly efficient signal transmission.

ATP transport by VNUT is allosterically activated by low concentrations of Cl^−^, peaking at 5–20 mM, but is strongly inhibited at higher concentrations. While Cl^−^ is not essential for transport, it serves as a regulatory factor (Moriyama et al., 2025). Similar Cl^−^-dependent activation is observed in vesicular glutamate transporters (VGLUTs encoded by *SLC17A6, A7, and A8*), which, like VNUT, belong to the SLC17 family. In these transporters, Cl^−^ conductance allows Cl^−^ to permeate the protein, thereby regulating the ON/OFF state of L-glutamate transport in response to changing Cl^−^ concentrations during synaptic vesicle recycling and enabling vesicles to be refilled with L-glutamate (Eriksen et al., 2020; Piet et al., 2020). It is believed that ATP transport by VNUT is regulated through a mechanism similar to that in VGLUTs (Moriyama et al., 2025).

VNUT plays a critical role in ATP exocytosis. In mice lacking the *Slc17a9* gene, ATP is not loaded into secretory vesicles, resulting in the absence of exocytosed ATP (Sakamoto et al., 2014) (Figure 1D). Reduced *SLC17A9* expression results in a proportional decrease in exocytosed ATP (Kabashima et al., 2026), whereas rescue of VNUT expression in *Slc17a9*-deficient mice restores spinal extracellular ATP levels (Masuda et al., 2016). As will be discussed later, *SLC17A9* knockdown or knockout, VNUT inhibitors, and V-ATPase inhibition all diminish ATP exocytosis. Notably, in VNUT knockout mice, exocytosed ATP is abolished, whereas channel-mediated eATP production remains unaffected, enabling a clear quantitative assessment of its contribution.

### 2.2 Vesicular substrates and associated molecules of VNUT

ATP-filled granules contain a diverse array of substances beyond ATP. These include nucleotides such as GTP, UTP, UDP, ADP, diadenosine polyphosphate (Ap(n)A), and β-nicotinamide adenine dinucleotide (βNAD^+^); inorganic ions such as inorganic phosphate and inorganic polyphosphate (PolyP); neurotransmitters such as 5-HT and acetylcholine; peptide hormones such as insulin; and acidic proteins such as chromogranins (Zimmermann, 1994; Winkler et al., 1982; Estevez-Herrera et al., 2016a). The relationship between these substances and VNUT is discussed below.

#### 2.2.1 VNUT substrates

VNUT is a polyspecific anion transporter responsible for moving a variety of organic and inorganic anions. In addition to ATP, ATP-filled granules frequently contain other nucleotides, including GTP, UTP, UDP, ADP, AMP and Ap(n)A. The specific composition of these nucleotides within vesicles may reflect the substrate specificity of VNUT (Sawada et al., 2008). VNUT preferentially recognized ATP, GTP, UTP and ADP as transport substrates, whereas AMP, adenosine and adenine recognized much less efficiently (Sawada et al., 2008; Larsson et al., 2012). Upon exocytosis, these nucleotides participate in purinergic chemical transmission (Figures 1B,C).

UTP and UDP are endogenous purinoceptor agonists and are significant pyrimidine nucleotides implicated in the development of pain and inflammation. However, their secretory mechanisms have remained largely unclear. Intracellular concentrations of UTP and UDP are estimated to be around 0.5–8 nmol per 10^7^ cells (Lazarowski et al., 1999), and some studies indicate their release may occur via permeation through anion channels (Chekeni et al., 2010). In contrast, secretory granules such as chromaffin granules can contain up to 10 mM (Winkler et al., 1982). VNUT transports UTP with about 20% of the efficiency observed for ATP. UTP is believed to be exocytosed alongside ATP and can bind to P2Y4 receptors with an EC_50_ of approximately 0.14 μM (Attah et al., 2020).

Adenosine, a substrate weakly recognized by VNUT, is mainly supplied in the extracellular space through ectonucleotidase-mediated degradation of ATP and ADP. In inflammatory cells such as macrophages, adenosine is known to exert anti-inflammatory effects (Cohen et al., 2013; Hasko et al., 1996; Hasko et al., 2000; Csoka et al., 2007).

#### 2.2.2 Candidate VNUT substrates

βNAD^+^ is a dinucleotide consisting of nicotinamide mononucleotide and AMP, serving as an essential coenzyme in intracellular metabolism. Beyond its intracellular role, βNAD^+^ can also be released into the extracellular space, where specific ectonucleotidases metabolize it into nicotinamide mononucleotide (NMN), ADP-ribose, cyclic ADP-ribose, and nicotinamide adenine dinucleotide phosphate (NADP^+^). These metabolites then participate in signal transmission via P2Y and P2X receptors (Moreschi et al., 2006; Haag et al., 2007; Fruscione et al., 2011; Pliyev et al., 2014; Mutafova-Yambolieva and Durnin, 2014). Recent studies have summarized the purinoceptor-mediated effects of βNAD^+^ (Durnin et al., 2020; Rivas-Yanez et al., 2020; Gasparrini et al., 2021). Additionally, multiple reports have highlighted the association between purinoceptor-mediated βNAD^+^ signaling and various biological functions (Audrito et al., 2021). Although βNAD^+^ is commonly believed to originate from the leakage of damaged cells, evidence suggests it may also be actively released via exocytosis, similar to other nucleotides. Further research is required to elucidate these mechanisms.

PolyP is a high-energy molecule composed of several hundred to a thousand orthophosphate units linked linearly. It is believed to be present in all organisms, from bacteria to mammals. In bacteria, PolyP acts as both an energy and phosphate source, and it plays diverse roles such as forming cellular structures, acting as a coenzyme, and serving as a signaling molecule in response to external stress. In human platelet dense granules, PolyP coexists with ATP, ADP and 5-HT (Muller et al., 2009). Subsequent studies revealed that ATP and PolyP also coexist in VNUT-containing vesicles—considered secretory lysosomes in astrocytes—and that they are released to participate in purinoceptor-mediated signaling (Holmstrom et al., 2013; Angelova et al., 2018). Kamila Nebesnaya *et al.* demonstrated through molecular docking that PolyP can bind to multiple P2X receptors, and showed that PolyP-induced increases in intracellular Ca^2+^ concentrations are inhibited by P2X receptor antagonists (Nebesnaya et al., 2024). Additionally, intracellular PolyP levels have been found to be elevated in astrocytes derived from mouse and patient induced pluripotent stem cells (iPSCs) carrying amyotrophic lateral sclerosis- or frontotemporal dementia-linked mutations, and PolyP levels are also increased in astrocyte-conditioned media from these cells (Arredondo et al., 2022). Although much remains unknown about the origin of PolyP, Andrey Abramov and colleagues showed that mitochondrial F0F1 transports H^+^ by degradation of PolyP, and synthesizes PolyP coupled with the respiratory chain (Baev et al., 2020).

PolyP, βNAD^+^, and their analogues (e.g., NAD-ribose) have been identified in certain ATP-secretory vesicles. These compounds—including ATP—all originate from mitochondria and share output, input, and termination mechanisms with purinergic chemical transmission. Although there is currently no evidence that VNUT transports these compounds, its multi-substrate recognition capability suggests it may fulfill this role. As the significance of βNAD^+^ and PolyP as intercellular signaling molecules becomes increasingly clear, identifying the vesicular transporter(s) responsible for their uptake into secretory vesicles and mitochondria is an urgent priority.

#### 2.2.3 Co-stored non-substrate molecules

VNUT-containing organelles, particularly synaptic vesicles and secretory granules in neuroendocrine cells, store not only ATP but also neurotransmitters such as 5-HT, noradrenaline, and L-glutamate, along with peptides like insulin and GLP-1, which themselves are not VNUT substrates (Estevez-Herrera et al., 2016a). These substances are co-released with ATP in response to specific stimuli. Notably, vesicular ATP has been shown to modulate the secretion of these co-released molecules. In other words, vesicular ATP can increase the concentration of co-existing neurotransmitters, thereby enhancing the chemical signaling output (quantum release). Monoamines such as 5-HT and noradrenaline are transported into vesicles by vesicular monoamine transporters through exchange with two H^+^. Theoretically, monoamines can be concentrated approximately 20,000-fold within the vesicle when the inside acidic pH gradient (ΔpH) is 1.5 and the inside positive Δψ is 80 mV, but actual concentrations can reach up to 100,000-fold (Omote et al., 2011). This heightened accumulation is partly due to the transport of MgATP into vesicles, which generates a synergistic effect through the electrochemical potential difference of H^+^, further increasing monoamine uptake (Bankston and Guidotti, 1996). As will be discussed later, the function of vesicular ATP as a biological hydrotrope is another mechanism that promotes the aggregation of peptide hormones and other substances coexisting within the vesicles (Patel et al., 2017). In chromaffin cells from *Slc17a9* knockout mice, vesicular monoamine transporter activity remains comparable to that in wild-type mice, yet both monoamine content and secretion are significantly decreased (Sakamoto et al., 2014). Ricardo Borges and colleagues demonstrated that both monoamine levels and quantum release from chromaffin granules declined following *SLC17A9* knockdown, while increased *SLC17A9* expression led to elevated monoamine and quantum release from these granules (Estevez-Herrera et al., 2016b).

### 2.3 Assays for VNUT activity

In studies of cellular ATP release, ATP concentrations in the cell supernatant are typically measured following stimulation. However, these measurements cannot distinguish between exocytosed ATP and that which originates from cell leakage or channel-mediated transport. To confirm ATP exocytosis specifically, methods are generally categorized into optical approaches using fluorescent labeling and assays of transporter activity.

MANT-ATP is a highly effective fluorescent ATP derivative that selectively labels ATP-filled vesicles when incubated with cells at physiological temperatures. Although the mechanism by which MANT-ATP labels ATP-filled vesicles was initially unclear, subsequent studies have confirmed that MANT-ATP is a suitable transport substrate for VNUT (Moriyama et al., 2025). MANT-ATP binds to the ATP-binding site of VNUT and competitively inhibits ATP transport. Reducing intracellular *SLC17A9* expression or activity prevents the accumulation of MANT-ATP within vesicles (Inoue et al., 2014; Nomura et al., 2025). These findings suggest that MANT-ATP, once transported into the cell via transporters such as ENT1/2, is actively taken up into vesicles by VNUT. On the other hand, caution is necessary when using acridine-based fluorescent dyes, such as quinacrine and acridine orange, which were frequently used to stain ATP-filled vesicles *in vivo*. The presence of these dyes in ATP-filled vesicles does not reflect the presence of ATP. Instead, the acidic environment inside ATP-filled granules, maintained by V-ATPase, causes the non-ionized (lipid-soluble) form of the amphiphilic amine acridine-based dye to bind to H^+^ within the granules and become ionized (water-soluble). As a result, the dye is distributed according to the H^+^ concentration gradient across the vesicle membrane, not the ATP concentration gradient (Schuldiner et al., 1972; Moriyama, 1996). Therefore, the degree of dye enrichment reflects the pH gradient (H^+^ concentration gradient) rather than the ATP concentration gradient (Hasuzawa et al., 2021a).

Biochemical measurements of VNUT activity are crucial for fully understanding its functional properties. Traditionally, ATP transport has been assessed using ATP-filled granules isolated from cells. However, these granules typically maintain a Donnan-type ion equilibrium due to the presence of membrane-impermeable acidic components, such as acidic proteins and high-molecular-weight polysaccharides. This equilibrium leads to an acidic internal pH and a Δψ (inside negative), which inhibit ATP transport. Additionally, the high concentration of ATP inside the vesicles complicates accurate measurement of active ATP transport. Consequently, reliable assessments of active ATP transport in ATP-filled vesicles have been extremely limited to date. To overcome these challenges, a method utilizing VNUT-containing proteoliposomes was developed (Sawada et al., 2008). In this technique, an ionophore establishes a diffusion potential across the liposome membrane, resulting in a positive internal membrane potential. ATP is then electrophoretically transported into the liposome, and the amount of uptake is quantified. While this method provides clear results, it is labor-intensive and has not been widely adopted. Moreover, it remained uncertain whether the properties of VNUT observed in liposome-based systems accurately reflected its transport activity in living organisms. Recently, a simpler approach using chromaffin granule membrane vesicles has been introduced (Moriyama et al., 2025) (Figure 1E). This method leverages VNUT’s capacity to transport sulfate. When chromaffin granule vesicles are incubated with MgATP and radioactive sulfate in a suitable solution, radioactive sulfate is taken up into the vesicles in response to the Δψ generated by the vacuolar H^+^-ATPase. Measuring this uptake provides a straightforward and user-friendly assay for VNUT activity.

This method revealed the characteristics of ATP transport by VNUT at the membrane vesicle level, uncovering novel properties that were previously undetectable with conventional techniques. Notably, VNUT was found to be driven by Δψ and to actively transport inorganic oxyanions, such as inorganic phosphate, in a Na^+^-independent fashion. Because ATP-containing vesicles consistently harbor a certain amount of phosphate, the source of this phosphate is of interest; these findings suggest that VNUT mediates the uptake of inorganic phosphate from the cytoplasm into vesicles. Following exocytosis, phosphate released from the vesicles is thought to be reabsorbed into the cell by type II and type III Na^+^-dependent phosphate transporters. As with VGLUT (Cheret et al., 2021), VNUT may contribute to the phosphate cycle *in vivo*.

### 2.4 VNUT as a protein marker for ATP exocytosis

ATP accumulates in organelles containing VNUT, making cells that express VNUT highly likely to secrete ATP via exocytosis. In reported cases to date, no clear exceptions to this association have been identified. As a result, VNUT expression is considered a reliable cellular and organellar marker for exocytosed ATP production. For instance, VNUT is found at high concentrations in gap junction vesicles of lateral giant fibrils in crayfish, indicating active exocytosed ATP production in this region (Ohta et al., 2011). VNUT has been identified in many cell types derived from all germ layers (ectoderm, mesoderm, and endoderm) and is localized in intracellular secretory vesicles, secretory lysosomes, and other unidentified vesicles. Table 1 summarizes the organs/tissues and cell types expressing VNUT as of 2017 (Moriyama et al., 2017), along with newly identified VNUT-containing organelles reported over the past decade.

**TABLE 1:** Expression, localization of VNUT and secretory stimulants of exocytosed ATP.

Organ (tissue)	Cell type (vesicle)	Stimulant of exocytosed ATP	References
Brain	Neuron (synaptic vesicle)	High K^+^, Ca^2+^ ionophore, action potentials	Larsson et al. (2012), Sakamoto et al. (2014), Ho T. et al. (2015)
​	Astrocyte (secretory lysosome and non-lysosomal vesicle)	Extracellular ATP (1 mM), L-glutamate (1 mM), Hypoxia (KCN, 4 mM), Ca^2+^ ionophore, Electrical Stimulation (Ca^2+^ wave propagation)	Zhang et al. (2007)
​	​	Ca^2+^ ionophore, extracellular ATP (300 μM), L-glutamate (1 mM)	Oya et al. (2013)
​	​	Hypoxic condition	Angelova et al. (2015)
​	​	Lipopolysaccharide (LPS)	Li et al. (2025)
​	Ventral brain stem astrocyte	Acidification, ATP (10 μM, 100 μM)	Kasymov et al. (2013)
​	Microglia	Ca^2+^ ionophore, extracellular ATP, non-hydrolyzable ATP analog (ATPγS), LPS	Imura et al. (2013)
​	​	Methylmercury (0.01 µM–0.1 µM)	Shinozaki et al. (2014)
​	​	Extracellular ATP/ADP, LPS	Li et al. (2025)
​	​	H_2_O_2_	Li et al. (2019)
Spinal cord (dorsal root ganglion)	Neuron	​	Nishida et al. (2014)
​	​	Peripheral Nerve Injury, Endoplasmic reticulum Ca^2+^ release	Masuda et al. (2016)
Peripheral nervous system (sciatic nerve)	Schwann cell (lysosomal vesicle)	Ammonium chloride (4 mM), extracellular ATP, nerve injury (Wallerian degeneration)	Shin et al. (2012)
(carotid body)	chemoreceptive type I cells	hypoxia, hypercapnia, acidification	Yokoyama et al. (2024)
Eye retina	Photoreceptor layer, Inner nuclear layer, Ganglion cell layer	​	Vessey and Fletcher (2012)
​	Photoreceptor cell, ganglion cell, inner nuclear layer, inner plexiform layer, outer plexiform layer	Potassium chloride, intraocular pressure, glaucoma progression	Perez de Lara et al. (2015)
​	Photoreceptor cell, bipolar cell, amacrine cell, astrocyte and Müller cell (retinal glia)	Light (Photosignals), Ca^2+^	Moriyama and Hiasa (2016)
Cornea and conjunctiva	Epithelium	Hyperosmotic Stress	Guzman-Aranguez et al. (2017)
Tongue (taste bud)	Type II taste cell	depolarization	Iw et al. (2009)
Parotid gland	​	​	Hiasa et al. (2014a)
Tooth	Odontoblast	Thermal Stimulation	Ikeda et al. (2016)
​	Periodontal ligament fibroblast	Mechanical stimulation	Mizuhara et al. (2020)
Adrenal gland	Chromaffin cell (chromaffin granule), PC12	Potassium chloride (50 mM): depolarization, intracellular Ca^2+^	Sawada et al. (2008)
Pancreas (islets of Langerhans)	α Cell (glucagon-containing secretory vesicle)	​	Sakamoto et al. (2014)
​	β Cell (insulin granule)	Glucose (20 mM)	Sakamoto et al. (2014)
​	​	Potassium chloride (50 mM), glucose (16.8 mM, 25 mM)	Geisler et al. (2013)
​	Acinar cell (zymogen granule)	Acetylcholine, cholecystokinin	Haanes and Novak (2010)
Airway	Epithelial goblet-like Calu-3 cell (mucin containing vesicle)	Intracellular Ca^2+^	Sesma et al. (2013), Kreda et al. (2007)
​	bronchial epithelial cell	Hypotonic stimulation, sterile supernatants of mucopurulent material from cystic fibrosis lungs (inflammatory environment), thrombin, shear stress and compressive stress	Okada et al. (2013), Hu et al. (2017)
Lung	Lung cancer A549 cell	Transforming growth factor-β1 (TGF-β1)	Takai et al. (2012)
Esophagus (esophageal mucosa)	Esophageal keratinocyte	Chemical, heat, stretch	Mihara et al. (2011)
​	​	​	Wolf-Johnston et al. (2012)
Stomach (muscle)	Musculomotor neuron	​	Chaudhury et al. (2012)
Intestine	L Cell (GLP-1/PYY-containing vesicle)	Potassium chloride (55 mM)	Harada and Hiasa (2014), Lu et al. (2019)
Urinary bladder	Urothelial cell	Mechanical stretch	Nakagomi et al. (2016)
Liver	Biliary epithelial cell	​	Sathe et al. (2011)
​	Hepatocyte	Glucose	Tatsushima et al. (2021)
​	Hepatic stellate cell	Ca^2+^ ionophore (ionomycin), thapsigargin, serotonin	Kabashima et al. (2026)
Blood	Platelet (dense granule) (human platelet and MEG-01 cell)	​	Hiasa et al. (2014b)
Immune system	Neutrophil	​	Harada et al. (2018)
​	Macrophage: THP1 cell, RAW 264.7 cell	LPS, vesicular stomatitis virus (VSV) infection	Sakaki et al. (2013), Kato et al. (2017), Zhang et al. (2017)
​	T Cell (CD4) (mouse, human T cell lymphoma Jurkat cell)	​	Tokunaga et al. (2010)
​	T helper 1	CD3/CD28 ligand (T cell activation)	Wu et al. (2025)
Electric organ (Torpedo)	Electromotor neuron (synaptic vesicle)	​	Li and Harlow (2014)
Cray fish	Lateral giant fiber	​	Ohta et al. (2011)
Kidney and muscle	Human embryonic kidney cell 293 Mouse myoblast C2C12 cell Monkey kidney COS1 cell (secretory lysosome)	​	Cao et al. (2014)
Skin	Keratinocytes	​	Inoue et al. (2014)
​	​	*C. albicans and C. albicans*-derived soluble β-glucan	Maruyama et al. (2018)
​	​	Nociceptive stimuli (capsaicin, potassium hydroxide)	Reno et al. (2024)

As discussed above, secretory vesicles containing VNUT frequently also store various signaling molecules alongside ATP. For example, in the pancreatic islets, VNUT is present in glucagon granules of α cells and insulin granules of β cells (Sakamoto et al., 2014), suggesting that ATP is exocytosed in coordination with insulin and glucagon release. The specific role of exocytosed ATP in the islets of Langerhans will be addressed later. In intestinal L cells, VNUT is localized to GLP-1-containing secretory granules, indicating that ATP and GLP-1 are co-secreted (Harada and Hiasa, 2014). Supporting this, Lu et al. showed that L cells release both ATP and GLP-1 in response to GLP-1 secretion-promoting substances, using mouse-derived GLUTag cells and mouse intestinal L cells. They concluded that the released ATP acts in a paracrine fashion on intestinal epithelial cells and afferent nerve fibers (Lu et al., 2019). Notably, dual-labeled immunohistochemistry reveals that VNUT and GLP-1 do not always colocalize, but there is a high degree of overlap with peptide YY (PYY). Ultimately, a thorough understanding of the signal transmission mechanisms of coexisting molecules will enable predictions about information transfer via exocytosed ATP. Conversely, insights into ATP exocytosis can shed light on the secretion mechanisms of coexisting signaling molecules.

### 2.5 Critical summary

Taken together, the evidence reviewed in this section establishes VNUT as the core molecular determinant of vesicular ATP loading and, therefore, of ATP exocytosis. This conclusion is supported by convergent biochemical, genetic, and rescue data. At the same time, the molecular framework remains incomplete. VNUT is clearly polyspecific, but the physiologically relevant substrate spectrum beyond ATP is not yet fully resolved, and proposed cargoes such as βNAD+ and polyphosphate remain candidates. Although VNUT expression is a useful marker of capacity for ATP exocytosis, direct functional assays remain essential in complex tissues where vesicular, conductive, and leakage-mediated ATP release may coexist.

## 3 Stimuli triggering for ATP exocytosis

ATP stored in intracellular vesicles by VNUT is released as exocytosed ATP in response to various stimuli and thereby participates in signal transduction. An increase in intracellular Ca^2+^ concentration represents the final trigger for ATP exocytosis. Ionomycin, a Ca^2+^ ionophore, is reported to induce ATP exocytosis in many cell types (Inoue et al., 2014; Kreda et al., 2007; Imura et al., 2013; Zhang et al., 2007). The suppression of eATP release by Ca^2+^ chelation (Kabashima et al., 2026; Li et al., 2025; Li et al., 2019; Kato et al., 2017; Harada et al., 2018) or low temperature (Kabashima et al., 2026; Harada et al., 2018), indicates the involvement of an exocytotic mechanism. Treatment with the L-type Ca^2+^ channel blocker nilvadipine during *in vitro* T helper (T_H_) 1 cell differentiation abolishes the differences in IFN-γ–positive cell proportions between wild-type and CD4^+^ T cell–specific *Slc17a9* knockout cells (Wu et al., 2025). Thapsigargin, which induces Ca^2+^ release from the endoplasmic reticulum, also triggers ATP exocytosis (Kreda et al., 2007). Intriguingly, thapsigargin is reported to upregulate *SLC17A9* (Kabashima et al., 2026), suggesting that Ca^2+^ signaling regulates VNUT at both functional and expression levels.

In *Slc17a9* knockout CD4^+^ T cells, CD3^−^and CD28-induced Ca^2+^ influx is reduced compared with that in wild-type cells (Wu et al., 2025). In astrocytes, the ecto-ATPase apyrase reduces intracellular Ca^2+^ elevation in response to poly (I:C). These findings suggest that exocytosed ATP is involved in stimulus-induced Ca^2+^ signaling (Beckel et al., 2018).

*In vivo,* various stimuli induce VNUT-mediated ATP exocytosis. Representative stimuli are summarized below in five categories.

### 3.1 Pathogen-associated molecular patterns (PAMPs)

Pathogen-associated molecular patterns (PAMPs) are molecular patterns carried by exogenous pathogens that trigger innate immune responses through pattern-recognition receptors (PRRs) (Li et al., 2024).

In response to PAMPs, a variety of cell types, including both immune and parenchymal cells, have been shown to exocytose ATP, thereby contributing to early host-defense responses. Lipopolysaccharide (LPS), a representative PAMP, induces ATP exocytosis in macrophages (Kato et al., 2017; Sakaki et al., 2013), microglia, astrocytes (Li et al., 2025), and urothelial cells (Silberfeld et al., 2020). In an LPS-induced brain inflammation model, ATP/ADP release was increased and significantly attenuated in whole-body *Slc17a9* knockout mice, whereas astrocyte- or microglia-specific conditional knockout each produced only partial reductions, indicating that VNUT in both astrocytes and microglia contributes to LPS-induced ATP/ADP release (Li et al., 2025).

Similarly, *Candida albicans*-derived soluble β-glucan (CSBG) and *C. albicans* induce ATP release from keratinocytes, and the CSBG-induced response is markedly reduced in *Slc17a9*-deficient cells (Maruyama et al., 2018); the TLR3 agonist poly (I:C) induces lysosomal ATP exocytosis in astrocytes and retinal pigment epithelial (RPE) cells (Beckel et al., 2018); vesicular stomatitis virus (VSV) infection induces ATP exocytosis in macrophages (RAW 264.7 cells) (Zhang et al., 2017); and HIV-1 gp120 induces lysosomal exocytosis and ATP release in Schwann cells, and these effects are blocked by clodronate pretreatment (Datta et al., 2019). Collectively, these findings indicate that VNUT plays an important role in host defense against bacterial, fungal, and viral pathogens.

### 3.2 Physical and chemical stimuli

Diverse sterile physical and chemical stimuli have also been shown to induce ATP exocytosis. Nociceptive stimuli such as capsaicin and potassium hydroxide (KOH) induce the release of ATP-containing microvesicles from human keratinocytes, and this response is attenuated by clodronate (Reno et al., 2024). Changes in hydrostatic pressure stimulate ATP release from the uroepithelium, and this release is partially inhibited by inhibitors of vesicular transport, supporting a vesicular component (Wang et al., 2005). In an overactive bladder model induced by partial bladder outlet obstruction, bladder *Slc17a9* mRNA and protein expression are significantly increased at 4 weeks (Huang J. et al., 2023). Hypotonic stimulation promotes ATP release in primary human bronchial epithelial cells, and in inflamed airway epithelia this response is enhanced via Ca^2+^-dependent vesicular pathways (Okada et al., 2013; Hu et al., 2017). Oxidative stress induced by H_2_O_2_ triggers ATP release via Ca^2+^-dependent lysosomal exocytosis in astrocytes (Li et al., 2019). In the carotid body, chemoreceptive type I cells that monitor arterial pO_2_, pCO_2_, and pH express VNUT and P2Y12 purinoceptors (Yokoyama et al., 2024), and hypoxia depolarizes these cells and triggers ATP release that is inhibited by L-type Ca^2+^ channel blockers and reduced by apyrase (Buttigieg and Nurse, 2004). Hypoxic stimulation also induces exocytosis of VNUT-containing vesicles in astrocytes (Angelova et al., 2018). Chemical hypoxia induced by potassium cyanide (KCN) has been shown to induce lysosomal ATP exocytosis in cultured astrocytes and acute hippocampal slices (Zhang et al., 2007).

In urothelial cells and airway epithelia, conductive pathways also contribute to eATP release, with evidence for connexin hemichannels in the uroepithelium (Wang et al., 2005) and pannexin one in airway epithelia (van Heusden et al., 2021), suggesting complex cross-talk with the nucleotide-induced ATP exocytosis discussed below.

### 3.3 Neurological stimuli

ATP is an important signaling molecule in the nervous system. It functions as a neurotransmitter in sensory neurons, motor neurons, and central nervous system neurons, where it can exert either excitatory or inhibitory effects. ATP is released not only from presynaptic terminals but also from postsynaptic membranes and glial cells, thereby contributing to neuron–glia communication (Fields and Burnstock, 2006). In a rat spinal nerve ligation model, cerebrospinal fluid ATP concentrations are increased, and this increase is suppressed by VNUT inhibition with Evans blue (Yin et al., 2019). Similarly, in a rat model of inflammatory and neuropathic pain induced by intra-articular injection of monoiodoacetate (MIA) into the knee joint, ATP concentrations in the cerebrospinal fluid are elevated 14 days after injury, together with increased *Slc17a9* expression in the ipsilateral dorsal horn of the spinal cord (Kwok et al., 2021). In another neuropathic pain model, chronic constriction injury of the sciatic nerve increases spinal VNUT expression, whereas intrathecal administration of botulinum toxin A reduces VNUT expression and exerts marked analgesic effects (Shi et al., 2023).

### 3.4 Endogenous signaling molecules

Some endogenous signaling molecules released by immune and non-immune cells also induce ATP exocytosis. *In vitro* stimulation of naive CD4^+^ T cells from wild-type mice with anti-CD3 and anti-CD28 antibodies promotes eATP release, whereas stimulated *Slc17a9*-conditional knockout T cells exhibit markedly lower eATP levels (Wu et al., 2025). Bafilomycin A1 treatment of Th1 cells decreases eATP levels and increases the proportion of IFN-γ-positive cells, indicating that ATP release following T-cell stimulation during Th1 differentiation negatively regulates IFN-γ production (Wu et al., 2025). Transforming growth factor-β1 (TGF-β1) induces eATP release in human lung cancer A549 cells, and this is suppressed by *SLC17A9* knockdown. Moreover, TGF-β1-induced cell migration and actin remodeling are attenuated in *SLC17A9* knockdown cells, suggesting that autocrine signaling via ATP exocytosis and P2 receptor activation is essential for amplification of TGF-β1-induced migration in lung cancer cells (Takai et al., 2012). TGF-β1 has also been reported to upregulate *SLC17A9* in hepatic stellate cells (Kabashima et al., 2026). In hepatic stellate LX-2 cells, serotonin induces VNUT-dependent ATP exocytosis, and this effect is blocked by clodronate. Glutamate induces ATP release via lysosomal exocytosis in cultured astrocytes and acute hippocampal slices (Zhang et al., 2007).

In well-differentiated primary human bronchial epithelial (WD-HBE) cells from both normal and cystic fibrosis donors, exposure to sterile supernatants of mucopurulent material enhances VNUT, but not pannexin 1, mRNA expression (Okada et al., 2013). Human airway epithelial Calu-3 cell cultures, which constitute a mixed population of non-mucous cells and mucin-secreting goblet-like cells, coordinately release ATP and mucin in a Ca^2+^-dependent manner (Kreda et al., 2007). Protease-activated receptor (PAR) agonists, including thrombin, stimulate the coordinated release of mucin and ATP from WD-HBE cells and Calu-3 cells. ATP, ADP and AMP are released into the mucosal compartment, and these adenine nucleotides likely contribute to paracrine signaling to ciliated cells, thereby promoting ion/water secretion and appropriate hydration of goblet cell-released mucins (Kreda et al., 2010). In alveolar type II cells (AT2), ATP is stored together with pulmonary surfactant in large secretory lysosomes termed lamellar bodies (LBs) via VNUT. Stimulation of LB exocytosis with UTP or PMA increases eATP (Fois et al., 2018). The secreted vesicular ATP then activates P2X4 receptors in an autocrine manner, inducing fusion-activated Ca^2+^ entry.

### 3.5 Extracellular nucleotide-induced ATP exocytosis

As described above, the fact that UTP increases eATP suggests nucleotide-induced ATP exocytosis. ATP-induced Ca^2+^-dependent lysosomal ATP exocytosis plays an important role in long-range microglial migration (Dou et al., 2012). ATP-induced increases in intracellular Ca^2+^ have been reported not only in mesenchymal stem cells (Jiang et al., 2017), macrophages (Zumerle et al., 2019), and T cells (Yip et al., 2009), but also in parenchymal cells including astrocytes (Suadicani et al., 2006), keratinocytes (Burnstock et al., 2012), urothelial cells (Chess-Williams et al., 2019), and hepatocytes (Senfeld et al., 2023). Expression of VNUT has been reported in these cell types, suggesting that nucleotide-induced ATP exocytosis may function as a signal amplification mechanism.

During the early phase of bladder filling, mild distension of the urothelium induces VNUT-dependent ATP release, which likely facilitates urine storage by inducing fusion of vesicles with the urothelial cell plasma membrane, thereby increasing the surface area available to accommodate the incoming urinary volume (Nakagomi et al., 2016). During the late phase of bladder filling, greater distension is thought to recruit additional ATP-release mechanisms, including mechanosensitive pathways involving TRPV4, PIEZO channels, and pannexin 1, thereby enhancing afferent firing and promoting the micturition reflex (Nakagomi et al., 2016; Mutafova-Yambolieva, 2025). These pathways are thought to be linked to distinct functions. Notably, VNUT expression in urothelial cells exhibits time-dependent/circadian variation, and ATP is concentrated in acidic vesicles (Nakagomi et al., 2016; Ihara et al., 2018). V-ATPase, a functional partner of VNUT, is also abundantly present in the luminal plasma membrane and endosome-like vesicles of urothelial cells, and the urinary acidification mediated by this pump shows diurnal variation (Tomochika et al., 1997).

A coordinated relationship between leakage- and exocytosis-derived eATP has also begun to emerge. Bone remodeling is a physiological process essential for maintaining bone structure and strength and is required for growth. Both leakage-derived extracellular ATP and exocytosed ATP are important in osteoblast responses to mechanical stimulation. Mechanical stimulation causes ATP release from osteoblasts predominantly through transient membrane injury, whereas Ca^2+^/PLC/PKC-dependent vesicular exocytosis facilitates membrane repair and thereby limits further ATP release (Mikolajewicz et al., 2018). Furthermore, during osteoblast differentiation under mechanical loading, VNUT-dependent exocytotic ATP release suppresses osteoblast differentiation (Inoue et al., 2020).

These findings suggest that leakage- or channel-mediated eATP supply and ATP exocytosis may cooperate via nucleotide-induced ATP exocytosis as part of a complex biological regulatory system.

### 3.6 Glucose as a metabolic trigger of ATP exocytosis

The stimuli described above—pathogen-associated patterns, physicochemical stimuli, neurological stimuli, endogenous signaling molecules, and extracellular nucleotides—all act as informational signals that promote Ca^2+^-dependent ATP exocytosis. By contrast, glucose acts as a metabolic signal and induces VNUT-mediated ATP exocytosis in several cell types. Glucose-induced responses are initiated by intracellular metabolic processing, which leads to changes in membrane excitability followed by Ca^2+^ influx.

The phenomenon whereby glucose elevates intracellular Ca^2+^ levels has long been recognized in pancreatic β-cells (Misler et al., 1992). An increase in intracellular ATP generated from glucose inhibits ATP-sensitive K channels, causing membrane depolarization. This, in turn, induces Ca^2+^ influx through voltage-dependent Ca^2+^ channels, increases intracellular Ca^2+^, and leads to sustained Ca^2+^ oscillations, ultimately triggering Ca^2+^-dependent exocytosis of insulin granules. In addition to glucose, stimulation with methyl pyruvate or leucine/glutamine rapidly increases ATP levels in the cytosol and mitochondria and also elevates Ca^2+^ (Tanaka et al., 2014). The facts that inhibition of ATP production suppresses the initial Ca^2+^ increase and that maintenance of cytosolic Ca^2+^ oscillations in pancreatic β-cells requires persistently high cytosolic ATP levels support the view that glucose acts as a metabolic signal via energy metabolism.

VNUT is localized on insulin granules in pancreatic β-cells, where ATP is co-stored with insulin and co-secreted in response to glucose stimulation through a VNUT-dependent mechanism (Sakamoto et al., 2014). High glucose stimulation promotes ATP secretion from islets of wild-type mice. In islets from *Slc17a9* knockout mice, glucose-responsive ATP secretion is absent, whereas insulin secretion is enhanced, suggesting that exocytosed ATP and/or its degradation products negatively regulates insulin secretion through purinergic feedback signaling. This interpretation is supported by the improved glucose tolerance of *Slc17a9* knockout mice. Thus, in pancreatic β-cells, the glucose–intracellular ATP production–Ca^2+^ elevation–ATP exocytosis axis provides one model of a metabolic signal linking the cellular energy state to signal transduction.

Additional functions have also been suggested for ATP co-storage in insulin granules. ATP is known to act as a biological hydrotrope that can prevent protein aggregation and dissolve pre-existing aggregates at physiological millimolar concentrations (Patel et al., 2017). In line with this idea, Panagiotou and colleagues reported in a preprint that VNUT-dependent ATP loading occurs at an early stage of insulin granule biogenesis and requires crosstalk with mitochondria (Panagiotou et al., 2026).

In hepatocytes, high glucose triggers VNUT-mediated ATP exocytosis. Primary cultured mouse hepatocytes secrete ATP in response to high glucose, followed by triglyceride secretion. In hepatocytes from *Slc17a9* knockout mice, glucose-induced ATP release is absent or markedly blunted, and the triglyceride response is suppressed. Triglyceride secretion from primary hepatocytes is inhibited by clodronate and by the P2Y receptor antagonists PPADS and MRS2211 (Tatsushima et al., 2021; Hasuzawa et al., 2021b). High glucose also induces ATP secretion in HepG2 hepatoma cells, and this response is inhibited by bafilomycin, clodronate, and monensin. In HepG2 cells, ATP and UTP activate P2Y2R-dependent Ca^2+^ signaling, suppress insulin-induced AKT phosphorylation, and promote gluconeogenesis (Senfeld et al., 2023).

Reports of glucose-induced ATP exocytosis are still limited, but in the type 2 diabetes model Otsuka Long-Evans Tokushima Fatty (OLETF) rat, bladder ATP release and *Slc17a9* expression are increased compared with those in control Long-Evans Tokushima Otsuka (LETO) rats (Kabuto et al., 2024). In streptozotocin-induced diabetic bladder dysfunction in male C57BL/6 mice, bladder VNUT and its mRNA expression increase during the compensated phase; VNUT remains elevated at 12 weeks, whereas the mRNA increase is not evident at 12 weeks (Yang et al., 2019). Further elucidation is needed regarding the relationship between glucose and ATP exocytosis in other organs.

In relation to glucose-induced ATP exocytosis, the following finding—that certain glucose metabolites suppress ATP exocytosis—is also of interest when considering metabolic signaling.

Phosphoenolpyruvate (PEP), a major intermediate metabolite in glycolysis, has an exceptionally high phosphate group transfer potential *in vivo* (−62 kJ/mol). PEP is normally generated in the cytosol during glycolysis, but it can also be synthesized from oxaloacetate by phosphoenolpyruvate carboxykinase. In particular, the mitochondrial isoform PEPCK-M generates mitochondrial PEP, which can then be exported to the cytosol (Stark and Kibbey, 2014). It has been shown that physiological concentrations of PEP competitively inhibit ATP transport by VNUT (ID_50_ = about 20 μM) (Moriyama et al., 2025). These findings suggest crosstalk between glucose metabolism and ATP exocytosis via PEP. PEP is a negative regulator of Th17 differentiation and also serves as a metabolic checkpoint in antitumor T-cell responses (Ho PC. et al., 2015). In differentiating Th17 cells, increased intracellular PEP levels, for example, after PEP supplementation or inhibition of downstream glycolytic enzymes, inhibit IL-17A expression. Mechanistically, PEP binds JunB and inhibits DNA binding of the JunB/BATF/IRF4 complex, thereby suppressing the Th17 transcriptional program (Huang T. Y. et al., 2023). These findings suggest a link between metabolic signals and endogenous signals involved in immune responses (Figure 1B).

### 3.7 Critical summary

These diverse stimuli can be unified under a common mechanism in which intracellular Ca^2+^ elevation serves as the final trigger, while upstream signals can be broadly classified into informational (e.g., PAMPs, neurotransmitters) and metabolic (e.g., glucose) inputs. The concept of metabolic control of ATP exocytosis links cellular energy state to purinergic output, thereby contributing to inflammatory responses. An additional conceptual point is the idea of nucleotide-induced ATP exocytosis, which may act as a feed-forward amplification mechanism to propagate local signals across tissues. Future studies will be required to clarify the context-dependent role of ATP exocytosis in vivo—whether as an initiating signal, a signal amplifier, or a feedback regulator.

## 4 Pharmacological manipulation of ATP exocytosis

### 4.1 Current pharmacological approaches to inhibit ATP exocytosis

Inhibiting vesicular ATP filling suppresses ATP exocytosis. Potential regulatory targets for ATP exocytosis include: (Lipmann et al., 1941) the transcription, translation, and intracellular transport of VNUT; (Holton and Holton, 1954) VNUT transport activity; (Burnstock, 1972); V-ATPase-driven proton transport; (Burnstock, 2007) the ion and proton permeability of the vesicular membrane; and (Abbracchio et al., 2009) the machinery involved in exocytotic membrane fusion, such as SNAREs (Figure 1F). The following sections will focus on targets 2 to 4.

Representative VNUT inhibitors include clodronate, a first-generation bisphosphonate, and Evans blue, a dye used for vital staining. Notably, clodronate exhibits high selectivity, with an ID_50_ of 15.6 nM for VNUT inhibition and minimal effects on other vesicular transporters, such as VGLUT (Kato et al., 2017; Hasuzawa et al., 2021b; Moriyama and Nomura, 2018). Supplementing the culture medium with 1 μM clodronate during cell culture can almost completely deplete vesicular ATP. Clodronate liposomes—a formulation encapsulating clodronate in liposomes—are commercially available as selective macrophage removal agents. Unlike free clodronate, however, these liposomes induce apoptosis via macrophage uptake and the mitochondrial toxicity of their metabolites.

Inhibiting V-ATPase with macrolide antibiotics such as bafilomycins and concanamycins neutralizes vesicular pH and also suppresses ATP exocytosis. However, these V-ATPase inhibitors are extremely cytotoxic. At concentrations exceeding several micromolar, they also inhibit P-type ATPases, such as Na^+^/K^+^-ATPase, confounding the interpretation of results and making it difficult to attribute observed effects solely to V-ATPase inhibition (Bowman et al., 1988). Unfortunately, such methodological oversights are common in the literature.

Dissipating the electrochemical proton gradient across the vesicular membrane using H^+^ conductors such as carbonyl cyanide 3-chlorophenylpydrazone (CCCP, a proton conductor) or lipophilic anions is another effective strategy for depleting ATP within vesicles. Numerous epidemiological studies have demonstrated that ω3 group polyunsaturated fatty acids (PUFAs) possess therapeutic and preventive effects on respiratory and cardiovascular diseases, metabolic disorders such as diabetes, immune system diseases, and neurological conditions. Research continues to advance in harnessing their potent health benefits and elucidating their mechanisms of action (Poudyal et al., 2011; Zhou et al., 2022). In the body, ω3 PUFAs are metabolized into resolvins and protectins, which exert anti-inflammatory effects via their respective receptors (Serhan and Levy, 2025). Additionally, PUFAs can translocate to the cell membrane as amphiphilic anions and modulate the function of ion channels and transporters by interacting directly with phospholipids and membrane proteins (Stillwell and Wassall, 2003; Bruno et al., 2013). Recent findings report that eicosapentaenoic acid (EPA) acts as an allosteric inhibitor of VNUT, thereby suppressing ATP exocytosis (Kato et al., 2022). Our study indicates that this inhibition is mediated by EPA’s direct action on vesicular membranes, increasing H^+^ permeability and indirectly reducing ATP transport (Moriyama et al., 2022). EPA also markedly inhibits the vesicular transport of L-glutamate, monoamines, and GABA in brain synaptic vesicles (Roseth et al., 1998). PUFAs have further been reported to exhibit ionophoretic properties and indirectly inhibit Na^+^-dependent glutamate transporters (Wang et al., 2024). EPA-containing supplements are widely used worldwide, and highly purified EPA preparations are recommended as cardiovascular risk-reduction agents in adults with hypertriglyceridemia undergoing statin therapy (Calder, 2017; Bhatt et al., 2019; Bays et al., 2016). Although the precise mechanism of action remains unclear, it is thought to be multifactorial—potentially involving reduced hepatic VLDL-TG synthesis and secretion, as well as anti-inflammatory, antioxidant, plaque-stabilizing, and antiplatelet effects. Additional studies are required to clarify how these mechanisms relate to the inhibition of ATP exocytosis.

Metformin and imeglimin are diabetes medications that generally cause few significant side effects, even when administered at doses of 1–2 g/day for several months. Imeglimin, in particular, lowers blood glucose by enhancing glucose-dependent insulin secretion from pancreatic β-cells and improving glucose metabolism in both the liver and skeletal muscle. Both drugs strongly inhibit intracellular vesicular ATP storage at extremely low concentrations (around 1 μM) (Nomura et al., 2025; Senfeld et al., 2023). While the specific target sites of metformin and imeglimin as vesicular ATP storage inhibitors remain unknown, they may modulate crosstalk between ATP-filled granules and mitochondria (Figure 1C).

### 4.2 Critical summary

Collectively, these data provide proof of principle that ATP exocytosis is pharmacologically tractable, but they also show that the current toolset remains limited. Clodronate, Evans blue, V-ATPase inhibitors, proton conductors, EPA, metformin, and imeglimin all support the concept that ATP exocytosis can be suppressed at multiple levels. EPA, metformin, and imeglimin are already used clinically in metabolic and related disorders. Further studies are warranted to clarify the relationship between their therapeutic effects and VNUT inhibition.

## 5 VNUT and diseases

### 5.1 VNUT in neurological and psychiatric disorders

The functions of VNUT in neurons and glial cells have been extensively investigated, with particular attention to its involvement in pain. We previously reviewed in detail the involvement of VNUT in inflammatory pain, nociceptive pain, mechanical allodynia, painful gastrointestinal disorders, and neuropathic pain (Hasuzawa et al., 2020). Subsequent studies have further elucidated the role of VNUT in pain.

In a monosodium iodoacetate (MIA)-induced knee joint injury model in rats, increased firing of peripheral joint afferents was accompanied by elevated ATP levels in the cerebrospinal fluid and increased *Slc17a9* expression in the ipsilateral spinal dorsal horn (Kwok et al., 2021). In a chronic constriction injury (CCI)-induced neuropathic pain model in rats, a single intrathecal injection of botulinum toxin type A (BoNT/A) produced significant analgesic activity, accompanied by reduced VNUT expression and decreased ATP content in the spinal cord (Shi et al., 2023). Conversely, overexpression of VNUT in the spinal cord markedly reversed the antinociceptive effect of BoNT/A, suggesting that BoNT/A is involved in neuropathic pain by regulating VNUT expression in the spinal cord.

EPA, an omega-3 (ω-3) polyunsaturated fatty acid abundant in fish oil, has been identified as a physiological inhibitor of VNUT. Intravenous administration of EPA significantly attenuated paclitaxel-induced neuropathic pain in wild-type mice (Kato et al., 2022). In humans, associations between polymorphisms in *SLC17A9* and pain-related disorders have been reported. Phantom tooth pain (PTP), a rare neuropathic pain condition that occurs after pulpectomy or tooth extraction, has been associated with the rs735055 polymorphism of the *SLC17A9* gene and rs3732759 of the *P2RY12* gene (Soeda et al., 2022). Carriers of the minor allele of rs735055 reportedly have high *SLC17A9* mRNA expression in the spinal cord, suggesting a link between elevated *SLC17A9* expression and PTP. A systematic review reported that, across two genome-wide studies of fibromyalgia syndrome, *SLC17A9* was among eight genes replicated between studies as containing differentially methylated sites (Fischer et al., 2023). VNUT has also been implicated in neurological disorders beyond pain. In progressive multiple sclerosis, an inflammatory and neurodegenerative disease of the central nervous system, *SLC17A9* was identified as one of three genes harboring differentially methylated regions that were consistent across two independent case–control cohorts of CD4^+^ T cells (Maltby et al., 2020).

Emerging evidence also suggests a role for VNUT in psychiatric disorders. Astrocyte-specific deletion of *Slc17a9* in adult mice leads to increased anxiety, depressive-like behaviors, and decreased motivation for reward, especially in females, without significant impact on food intake, systemic glucose metabolism, cognition, or sociability. Female *Slc17a9* GFAPKO mice displayed reduced center-zone entries, reduced sucrose preference consistent with anhedonia, and increased immobility time in the forced swimming test, compared with control *Slc17a9* f/f littermates. These alterations were accompanied by significantly decreased basal extracellular dopamine levels in the nucleus accumbens, a strong trend toward reduced evoked dopamine release, and significantly increased expression of monoamine oxidase A (Maoa), which is involved in dopamine metabolism (Huang et al., 2025). In genome-wide methylation analyses of treatment-resistant schizophrenia (TRS), defined as a non-response to at least two trials of antipsychotic medication with an adequate dose and duration, the *SLC17A9*-associated probe (cg17221813) was one of six methylation probes within gene coding regions that were selected for validation after being identified as differentially methylated between TRS and non-TRS patients; in the validation set, this probe yielded an ROC AUC of 0.86, indicating good discrimination (Lu et al., 2023). In the validation set, mean methylation signature levels for the *SLC17A9* probe were highest in non-TRS (55.60), followed by TRS (40.35), and lowest in healthy controls (23.00).

The findings support VNUT as a potential therapeutic target in pain, as indicated by the analgesic effects of the VNUT inhibitors clodronate (Kato et al., 2017) and EPA (Kato et al., 2022), as well as the close relationship between the analgesic effect of botulinum toxin and VNUT regulation (Shi et al., 2023). By contrast, in psychiatric disorders and neurological diseases other than pain, the current evidence remains largely limited to correlative observations, including links between *SLC17A9/Slc17a9* expression and neuropsychiatric phenotypes, and further studies are needed to establish whether VNUT is therapeutically actionable in these conditions.

### 5.2 VNUT in metabolic disorders

As discussed previously, VNUT has been implicated in inflammatory signaling in multiple immune cell types (Hasuzawa et al., 2020). More recent studies increasingly suggest that VNUT is also deeply involved in chronic metabolic inflammation, a pathophysiological process central to metabolic syndrome.

Glucose stimulates ATP exocytosis, and accumulating evidence indicates that VNUT is closely involved in metabolic dysregulation. Whole-body *Slc17a9* knockout mice exhibit enhanced insulin sensitivity and improved glucose tolerance (Sakamoto et al., 2014). In a large human cohort, although the mechanism remains unclear, the rs3746750 polymorphism in *SLC17A9* was associated with a reduced risk of gestational diabetes mellitus in the Caucasian subgroup (Ramos-Levi et al., 2022). Refeeding after fasting as well as high-fat diets increase VNUT expression in the liver (Senfeld et al., 2023; Tatsushima et al., 2021). Conversely, genetic deletion of *Slc17a9* enhances insulin-induced AKT phosphorylation in the liver (Sakamoto et al., 2014). When subjected to a high-fat diet, these mice exhibit increased hepatic lipid accumulation but reduced macrophage infiltration and fibrosis progression (Tatsushima et al., 2021). In contrast, pharmacological inhibition of VNUT with clodronate suppresses lipid accumulation, inflammation, and fibrosis under methionine–choline-deficient (MCD) and high-fat high-cholesterol (HFHC) diets (Hasuzawa et al., 2021b). Clodronate also inhibits VNUT-dependent ATP exocytosis from hepatic stellate cells and attenuates liver fibrosis (Kabashima et al., 2026). These findings suggest that VNUT may play an important role in metabolic syndrome-related pathophysiology. ATP, while functioning intracellularly as an energy currency, acts extracellularly as a prototypical damage-associated molecular pattern (DAMP), linking energy metabolism and inflammation (Zindel and Kubes, 2020). Indeed, in patients with poorly controlled type 2 diabetes, increased proportions of P2X7-positive peripheral blood mononuclear cells have been reported, and the percentages of CD39-positive cells were significantly associated with HbA1c levels (Garcia-Hernandez et al., 2011).

Ulcerative colitis, a chronic relapsing inflammatory bowel disease, has also been associated with metabolic syndrome (Janani et al., 2025). *SLC17A9* expression is elevated in lesion sites compared with non-lesion sites and healthy control tissue (WO2017126637A1; Japanese Patent No. 7038434 [JP7038434B2]). Notably, unlike IL-17A, whose expression decreases during remission, *SLC17A9* expression remains elevated even in remission-phase lesions, which may suggest involvement of purinergic signaling in disease relapse (Figure 1G).

It is noteworthy that antidiabetic drugs such as metformin and imeglimin suppress vesicular ATP release. Although metformin is widely used as a first-line treatment for type 2 diabetes, its mechanism of action remains incompletely understood. While high concentrations exceeding therapeutic levels have been shown to inhibit mitochondrial complex I and activate AMPK, the contribution of these effects to clinical efficacy remains debated. Senfeld et al. demonstrated that therapeutically achievable concentrations of metformin (10–30 μM) suppress high glucose-stimulated ATP secretion from hepatocytes, similarly to clodronate and bafilomycin, and that metformin-dependent improvements in glucose tolerance are abolished in P2Y2R-null mice (Senfeld et al., 2023). Furthermore, imeglimin, a recently approved antidiabetic agent developed based on metformin, reduced vesicular ATP storage and release in hepatic stellate cells at a clinically relevant concentration (10 μM), while attenuating immune cell infiltration and liver fibrosis *in vivo* (Nomura et al., 2025). These findings suggest that VNUT-dependent ATP exocytosis represents a promising therapeutic target in metabolic syndrome-related disorders, particularly in the context of chronic metabolic inflammation.

### 5.3 VNUT in cancer

Recent studies have demonstrated that VNUT, encoded by *SLC17A9,* plays important roles in cancer pathophysiology. Analyses of publicly available databases revealed significantly increased *SLC17A9* expression in 15 of 34 cancer types (LUAD, COAD, BRCA, KIRP, DLBC, HNSC, LAML, KIRC, LIHC, BLCA, READ, STAD, LUSC, THCA, UCEC), whereas decreased expression was observed in ACC tissues (Li J. et al., 2023). Conversely, *SLC17A9* was significantly downregulated in prostate cancer cells and tissues (Mi et al., 2021). Across multiple cancer types, high *SLC17A9* expression is associated with adverse survival outcomes, including shorter overall survival (OS), disease-specific survival (DSS), or progression-free survival (PFS). In the pan-cancer analysis, however, high *SLC17A9* expression was associated with longer OS in BRCA and with longer DSS and PFS in LUAD (Li J. et al., 2023). Several mechanisms have been proposed for the role of VNUT in tumor progression. Below, we discuss the roles of VNUT in cancer cell proliferation, cell-cycle progression, cell death, tumor migration invasion, tumor immunity, and drug sensitivity.

Knockdown of *SLC17A9* suppresses proliferation in hepatocellular carcinoma, lung cancer, osteosarcoma, and clear cell renal cell carcinoma (Li J. et al., 2023; Wu et al., 2020; Gao et al., 2023; Li W. et al., 2023). In hepatocellular carcinoma, *SLC17A9* knockdown reduces the expression levels of METTL3 and YTHDF1; it has been reported that METTL3 overexpression promotes tumor growth *in vivo* and *in vitro* (Kui et al., 2022). Analysis of genes co-expressed with *SLC17A9* indicates associations with PI3K/Akt and MAPK signaling pathways, transporter activity, and channel activity. Inhibition of BRD4, a driver of hepatocellular carcinoma proliferation, suppresses *SLC17A9* expression in hepatocellular carcinoma cells (Lin et al., 2022).

In NSCLC, *SLC17A9* knockdown suppresses proliferation in A549 and H1299 cells and appears to affect cell-cycle progression. High *SLC17A9* expression is associated with oncogenic signatures, including cytochrome P450-mediated drug metabolism, biological oxidations, and starch and sucrose metabolism (Gao et al., 2023). In osteosarcoma, *SLC17A9* is associated with MAPK, Hippo, focal adhesion, and TGF-β signaling pathways (Li J. et al., 2023). In clear cell renal cell carcinoma, *SLC17A9* overexpression promotes epithelial–mesenchymal transition (EMT) via upregulation of PTHLH (Li W. et al., 2023) and also promotes proliferation and migration through regulation of *KCNH1* expression (Ke et al., 2025).

VNUT is also implicated in cell viability and cell death. Its deficiency induces lysosomal dysfunction, leading to lysosomal storage and cell death (Huang et al., 2022). In contrast, in prostate cancer, *SLC17A9* knockdown suppresses apoptosis and enhances invasion and migration, whereas overexpression exerts opposite effects (Mi et al., 2021). In hepatocellular carcinoma, *SLC17A9* positively correlates with ferroptosis-related genes, and its knockdown reduces expression of *ACSL4, CISD1,* and *ATP5MC3* (Kui et al., 2022).

VNUT is also involved in tumor migration, invasion, and possibly metastasis. In lung cancer cells, TGF-β1-induced migration is inhibited by pretreatment with ectonucleotidases or P2 receptor antagonists, as well as by *SLC17A9* knockdown, suggesting that ATP exocytosis-mediated autocrine signaling amplifies migration (Takai et al., 2012). In clear cell renal cell carcinoma, *SLC17A9* knockdown upregulates E-cadherin and downregulates N-cadherin, Snail, and vimentin, suppressing invasion and migration, whereas overexpression has the opposite effect (Gao et al., 2023). *SLC17A9* expression is also higher in lymph node-positive tumors (N1) than in node-negative tumors (N0) (Ke et al., 2025). Conversely, in prostate cancer, *SLC17A9* expression is reduced in metastatic tumors, and its knockdown promotes invasion and migration (Mi et al., 2021).

Beyond direct tumor growth effects, VNUT also influences tumor immunity. Cancer cells can evade immune surveillance by engaging immune checkpoint pathways that suppress cytotoxic lymphocyte function (Pardoll, 2012).

In hepatocellular carcinoma, *SLC17A9* expression correlates with infiltration of CD4^+^ T cells, macrophages, and neutrophils, and with immune marker genes for multiple subtypes, including Tfh, Th1, Th17, M1/M2 macrophages, TAMs, and NK cells (Kui et al., 2022). In LUAD and LUSC, high *SLC17A9* expression was associated with immune infiltration and with the expression of immunoinhibitory genes such as *CTLA-4, PD-1, LAG3,* and *TIGIT* in LUAD and *A2aR, PD-1, CTLA-4*, and *TIGIT* in LUSC (Gao et al., 2023). Similar immune-related associations have been suggested in clear cell renal cell carcinoma, and highe*r SLC17A9* expression may also be associated with a better response to immunotherapy (Li W. et al., 2023).

A recent study on Th1 differentiation highlights the immunological significance of VNUT. VNUT-dependent ATP exocytosis in Th1 cells suppresses IFN-γ production. CD4^+^ T cells from CD4^+^ T cell–specific *Slc17a9* conditional knockout mice produce significantly higher levels of IFN-γ than those from wild-type mice. In a tumor model using MC38 cells, tumors were significantly smaller in knockout mice, and treatment with the VNUT inhibitor clodronate markedly suppressed tumor growth (Wu et al., 2025). These findings suggest that VNUT is a potential target for cancer immunotherapy.

Finally, drug sensitivity analysis revealed that *SLC17A9* expression was positively correlated with the half maximal inhibitory concentration (IC_50_) of anticancer agents such as vorinostat, asparaginase, chelerythrine, hypothemycin, PX-316, entinostat, acrichine, LDK-378, and imexon, and negatively correlated with the IC_50_ values of ibrutinib, afatinib, and sonidegib (Li J. et al., 2023; Li W. et al., 2023). In clear cell renal cell carcinoma cell lines, VNUT expression increases with increasing concentrations of vorinostat, consistent with a possible association between VNUT and vorinostat resistance (Li W. et al., 2023).

Most of the cancer-related literature to date remains correlative, based on altered *SLC17A9* expression and its associations with survival outcomes. However, several knockdown and knockout studies, as well as studies showing that VNUT inhibition suppresses tumor growth, support a mechanistic contribution of VNUT-related ATP exocytosis to tumor proliferation, migration, and antitumor immunity. The relationship between metformin-mediated inhibition of VNUT-dependent ATP exocytosis (Senfeld et al., 2023) and the reduced cancer risk suggested by epidemiological studies of metformin use (Decensi et al., 2010) is an intriguing topic for future investigation. Further studies are needed to clarify the therapeutic significance of ATP exocytosis as a target in cancer.

### 5.4 Critical summary

Although the type and depth of evidence vary across neurological and psychiatric disorders, metabolic disorders, and cancer, the available studies collectively suggest that VNUT-related ATP exocytosis may be involved in a broad range of pathological conditions. It is difficult to identify a single unifying feature across these disease categories. However, chronic inflammation may represent one shared pathophysiological basis linking at least some of these conditions. Further studies will be required to clarify the disease-specific roles of exocytosed ATP and whether common mechanistic links exist across these pathological contexts.

## 6 Discussion

The identification of the vesicular nucleotide transporter (VNUT/SLC17A9) has fundamentally transformed our understanding of extracellular ATP biology by providing a molecular basis for regulated ATP exocytosis. Unlike ATP release via membrane damage or conductive pathways, VNUT-dependent exocytosis represents a quantitatively controllable and stimulus-coupled mechanism**,** positioning it as a central node in purinergic signaling.

A key conceptual advance emerging from recent studies is that extracellular ATP should not be regarded as a single entity, but rather as a heterogeneous signaling pool defined by its mode of release. Leakage-derived ATP primarily functions as a danger signal (DAMP), rapidly mobilizing immune responses, whereas channel-mediated ATP release supports tonic signaling and mechanotransduction (Dosch et al., 2018). In contrast, VNUT-mediated ATP exocytosis is tightly regulated by intracellular Ca^2+^ dynamics and metabolic cues, enabling spatiotemporally precise signaling with high local concentrations**.** This distinction is particularly important because VNUT-dependent ATP release can be selectively manipulated without disrupting other ATP release pathways, as demonstrated in *Slc17a9* knockout models, thereby allowing direct interrogation of its physiological relevance (Sakamoto et al., 2014). Another important insight is that VNUT-mediated ATP exocytosis functions as an amplification and integration system for diverse stimuli**.** Although the upstream triggers are highly heterogeneous—including pathogen-associated molecular patterns, mechanical stress, neurotransmission, and metabolic signals such as glucose—they converge on intracellular Ca^2+^ elevation as a final common pathway. Moreover, the phenomenon of “nucleotide-induced ATP release” suggests the existence of a feed-forward purinergic signaling loop, in which initial ATP release activates purinoceptors to further enhance ATP exocytosis. This mechanism may serve to propagate signals over long distances and coordinate multicellular responses, particularly in tissues such as the urothelium, airway epithelium, and central nervous system.

From a metabolic perspective, VNUT-dependent ATP exocytosis represents a previously underappreciated link between intracellular energy status and intercellular communication. In pancreatic β-cells, ATP co-released with insulin exerts negative feedback on insulin secretion via purinergic signaling, highlighting a self-regulatory mechanism that fine-tunes hormone release (Sakamoto et al., 2014)**.** Similarly, in adipose tissue, liver, and immune cells, exocytosed ATP modulates inflammation, lipid metabolism, and cellular stress responses. These findings suggest that VNUT operates not merely as a transporter but as a metabolic signal converter**,** translating intracellular ATP availability into extracellular regulatory signals.

The therapeutic implications of targeting VNUT are particularly compelling. Because VNUT selectively governs exocytosed ATP without affecting leakage- or channel-mediated ATP release, it offers a unique opportunity to modulate purinergic signaling upstream of receptor activation. As discussed in this review, preclinical studies have demonstrated that VNUT inhibition attenuates pain, inflammation, and pathological remodeling in various disease models. Importantly, this strategy may avoid some of the limitations associated with purinoceptor antagonists, such as broad receptor distribution and functional redundancy. However, several challenges remain, including the need to clarify the long-term consequences of VNUT inhibition, its tissue-specific roles, and potential compensatory mechanisms in ATP release pathways.

Despite these advances, several critical questions remain unresolved. First, the full spectrum of VNUT substrates and their physiological relevance is not yet completely understood. Molecules such as βNAD^+^ and polyphosphate may share vesicular storage and release mechanisms with ATP, but their transport pathways remain unclear. Second, the interplay between VNUT-mediated exocytosis and other ATP release mechanisms requires further investigation, particularly in complex *in vivo* settings where multiple pathways coexist. Third, the role of VNUT in chronic metabolic diseases, including obesity and type 2 diabetes, warrants deeper exploration, especially in the context of tissue-specific signaling networks.

In conclusion, VNUT-mediated ATP exocytosis represents a distinct and highly regulated arm of purinergic signaling that integrates environmental, neuronal, and metabolic inputs. By bridging intracellular metabolism and extracellular communication, VNUT plays a pivotal role in coordinating physiological responses and disease processes. Targeting this pathway offers a promising and conceptually novel therapeutic strategy for inflammatory and metabolic disorders, but its successful translation will require a more comprehensive understanding of its systemic and context-dependent functions.

## Nomenclature

### General abbreviations

5-HT: 5-hydroxytryptamine (serotonin)
ADP: adenosine diphosphate
AMP: adenosine monophosphate
Ap(n)A: diadenosine polyphosphate
AT2: alveolar type II
ATP: adenosine triphosphate
**βNAD** ^**+**^: β-nicotinamide adenine dinucleotide
BoNT/A: botulinum toxin type A
**CD4** ^**+**^: cluster of differentiation 4
CSBG: *Candida albicans*-derived soluble β-glucan
DAMP: danger-associated molecular pattern
DSS: disease-specific survival
ΔpH: pH gradient
ΔΨ: membrane potential
eATP: extracellular adenosine triphosphate
EMT: epithelial–mesenchymal transition
ENT1/2: equilibrative nucleoside transporters 1 and 2
EPA: eicosapentaenoic acid
GLP-1: glucagon-like peptide-1
GTP: guanosine triphosphate
HFHC: high-fat high-cholesterol
IFN-γ: interferon-γ
KCN: potassium cyanide
KOH: potassium hydroxide
LBs: lamellar bodies
LPS: lipopolysaccharide
MANT-ATP: 2'-(or-3′)-O-(N-Methylanthraniloyl) adenosine 5′-triphosphate
MCD: methionine–choline-deficient
MIA: monoiodoacetate
NSCLC: non-small cell lung cancer
OS: overall survival
PAMPs: pathogen-associated molecular patterns
PAR: protease-activated receptor
PEP: phosphoenolpyruvate
PFS: progression-free survival
Pi: inorganic phosphate
PolyP: inorganic polyphosphate
PRRs: pattern-recognition receptors
PTP: phantom tooth pain
PUFAs: polyunsaturated fatty acids
PYY: peptide YY
RPE: retinal pigment epithelial
SLC17A9: solute carrier family 17 member 9 (VNUT)
TAMs: tumor-associated macrophages
Tfh: follicular helper T cells
TGF-β1: transforming growth factor-β1
Th1: T helper 1
TRS: treatment-resistant schizophrenia
UC: ulcerative colitis
UDP: uridine diphosphate
UTP: uridine triphosphate
V-ATPase: vacuolar H^+^-ATPase
VGLUT: vesicular glutamate transporter
VNUT: vesicular nucleotide transporter
VSV: vesicular stomatitis virus
WD-HBE: well-differentiated primary human bronchial epithelial

### Cancer types (The cancer genome atlas abbreviations)

ACC: Adrenocortical carcinoma
BLCA: Bladder Urothelial Carcinoma
BRCA: Breast invasive carcinoma
COAD: Colon adenocarcinoma
DLBC: Lymphoid Neoplasm Diffuse Large B-cell Lymphoma
HNSC: Head and Neck squamous cell carcinoma
KIRC: Kidney renal clear cell carcinoma
KIRP: Kidney renal papillary cell carcinoma
LAML: Acute Myeloid Leukemia
LIHC: Liver hepatocellular carcinoma
LUAD: Lung adenocarcinoma
LUSC: Lung squamous cell carcinoma
READ: Rectum adenocarcinoma
STAD: Stomach adenocarcinoma
THCA: Thyroid carcinoma
UCEC: Uterine Corpus Endometrial Carcinoma
